# Development of a scale to measure the psychological resources of grit in adults

**DOI:** 10.1111/nhs.12973

**Published:** 2022-08-14

**Authors:** Sarah E. Schimschal, Denis Visentin, Rachel Kornhaber, Tony Barnett, Michelle Cleary

**Affiliations:** ^1^ Centre for Rural Health, School of Health Sciences University of Tasmania Launceston Tasmania Australia; ^2^ School of Health Sciences University of Tasmania Sydney New South Wales Australia; ^3^ School of Nursing University of Tasmania Sydney New South Wales Australia; ^4^ School of Nursing, Midwifery & Social Sciences CQUniversity Sydney New South Wales Australia

**Keywords:** Delphi technique, goals, grit, psychological resources, scale development, staff development, GPRS

## Abstract

Grit, a personality trait characterized by  having passion and perseverance for long‐term goals, has been linked to increased performance, retention, and well‐being in various fields. In the 15 years since the original grit scale was published, many studies have investigated factors that promote grit. However, a scale has not yet been developed measuring characteristics that can contribute to higher levels of grit. This study aimed to develop a novel scale to measure the psychological resources of grit. The Delphi technique was used to obtain consensus from a national and international panel of academics and practitioners who are experts in grit and related constructs. A total of 30 participants rated 100 scale items over three rounds of online surveys. Experts agreed that items selected for the final scale (*n* = 20) were essential, with 85% rating them as important or very important. The scale, called the Grit Psychological Resources Scale, has promise as a helpful tool for practitioners involved in staff development and building capabilities that contribute to goal achievement.

**Key Points**

- A new scale, called the Grit Psychological Resources Scale (GPRS), has been developed to measure the psychological resources of grit in adults.
- A national and international expert panel of academics and practitioners agreed on 20 items to be included in the scale using the Delphi Technique.
- The GPRS can be used as a tool to identify interventions that strengthen personal characteristics associated with goal pursuit and achievement.

## 1 INTRODUCTION

Many have sought to understand which personal characteristics drive high performance and achievement (Dai & Sternberg, 2021). One emerging area of enquiry is grit, a characteristic purported to enable one to pursue ideas, projects, and goals consistently over extended periods whilst overcoming adversity and setbacks (Duckworth et al., 2007; Duckworth et al., 2019). People with high grit levels are reported to experience better performance and health outcomes across disciplines, professions, and cultures (Datu, 2021). In nursing and medicine there has been considerable interest in grit and the positive role it likely plays in reducing burnout and turnover intention and improving performance and well‐being (Cho & Kim, 2022; Dam et al., 2019; Jeong et al., 2019; Jumat et al., 2020; Terry & Peck, 2020). Studies have also investigated the factors that may precede, mediate, or moderate higher levels of grit (Schimschal et al., 2021). The extensive nomological network of characteristics that appear to play a role in achieving long‐term goals can be explained by the conservation of resources (COR) theory (Hobfoll, 1989).

The COR theory was developed to provide a rigorous framework for conceptualizing and studying stress. Hobfoll's (1989) model is based on the view that people are inclined to conserve and build valuable resources, and any perceived or actual threat to such capital can result in stress. Resources include objects (e.g., housing, clothing), personal characteristics (e.g., attributes, skills), conditions (e.g., status, health), and energies (e.g., time, money) that people value and thus seek to retain or develop in the presence or absence of stressors (Hobfoll, 1989). As such, the motivation to conserve resources will vary between individuals according to the appraised value and contextual circumstances (Hobfoll, 2001). Building on this theory, Halbesleben et al. (2014) suggest that resources encompass “anything perceived by the individual to help attain his or her goals” (p. 1338). Using this definition, it follows that many resources can facilitate motivational tendencies toward goal attainment, such as those associated with grit.

Previous research has linked various characteristics to higher levels of grit and, subsequently, improved outcomes. For example, grittier individuals exhibit higher levels of curiosity, self‐awareness, and self‐determination, which support commitment to one's interests (Dugan et al., 2019; Jin & Kim, 2017; Sheldon et al., 2015). Similarly, perseverance with goals has been linked to increased hardiness, resilience, self‐efficacy, growth mindset, and self‐regulation (Armstrong et al., 2018; Kannangara et al., 2018; Lovering et al., 2015). The growing body of literature identifying factors contributing to grit indicates an opportunity to develop programs that target underlying characteristics. However, to ensure the design and delivery of effective training, facilitators should first understand the current state of individual or team capability. This study helps to address this need by developing a novel scale that can provide information on characteristics that likely enable grit. Research on measuring the propensity for gritty behavior was anticipated to be of particular interest and benefit to a range of professionals, especially those involved in promoting staff development and achievement.

## 2 BACKGROUND

In a companion paper (Schimschal et al., 2022), a model for the psychological resources of grit is proposed. The model was informed by previous work by Duckworth (2016), who identified interest, purpose, practice, and hope as key assets to increase grit. Following on from this work, a further 16 attributes were identified that could aid in accruing the four psychological resources of grit. Table 1 summarizes expected behaviors exhibited by individuals possessing high levels of these attributes in the context of grit. As indicated, exhibiting strength in the attributes associated with interest and purpose improves one's motivation to learn, adapt, and grow from experiences continuously. These behaviors are thought to facilitate consistent interest in and thus the pursuit of valuable activities and goals. Equally, the attributes associated with practice and hope help individuals acquire the mindset to overcome challenges in developing expertise. This disposition enables one's perseverance with building capabilities to achieve goals.

**TABLE 1:** Expected behaviors from individuals with high levels of attributes associated with the psychological resources of grit

Psychological resource	Attribute	Individual behaviors contextualized for grit
Interest	Curiosity (Berlyne, 1954)	Pursues new experiences Embraces challenges and uncertainty Slow to lose interest
	Self‐awareness (Duval & Wicklund, 1972)	Often uses self‐reflection Analyzes choices and behavior Uses insights to learn and adapt behavior
	Courage (Rate et al., 2007)	Takes considered risks Not afraid to try new things Open to change and failure
	Patience (Schnitker, 2012)	Is patient when exploring new things Remains calm when learning is hard Can focus on complex problems
Purpose	Self‐determination (Deci & Ryan, 1985)	Has control over actions Motivates self to work hard Pursues meaningful goals
	Self‐concordance (Sheldon & Elliot, 1999)	Pursues activities because they are valuable Follows through with plans to gain experience Persists with goals to stay motivated
	Self‐compassion (Neff, 2003)	Can learn from negative experiences Tolerant of choices and direction Accepting of setbacks and failure
	Emotional intelligence (Salovey & Mayer, 1990)	Notices how emotions influence behavior Is aware of things that bring happiness Can control emotions and persist with goals
Practice	Hardiness (Kobasa, 1979)	Remains composed under learning pressure Feels in control of expertise development Keeps trying when things are hard
	Resilience (Carver, 1998)	Good at dealing with challenges Can overcome setbacks and failure Improves from adversity
	Self‐efficacy (Bandura, 1977)	Confident with achieving goals Likes to set hard goals Attributes failure to effort
	Flow (Csikszentmihalyi, 1990)	Goals slightly exceed capabilities Has a clear direction Can perform seamlessly
Hope	Goal orientation (Dweck, 1986)	Failure is a motivation Leans in to challenges and learning Uses feedback to improve
	Growth mindset (Dweck, 2006)	Works to change traits Ties success to hard work Failure is seen as opportunity
	Optimism (Abramson et al., 1978)	Does not take setbacks personally Can overcome problems Learns from the past
	Self‐regulation (Baumeister & Heatherton, 1996)	Can change behavior Usually avoids distractions Adjusts pace to be effective

There are scales to measure grit (Duckworth et al., 2007; Duckworth & Quinn, 2009) and related constructs such as the Passion Scale (Vallerand et al., 2003) and Persistence Scale (Howard & Crayne, 2019). However, a single scale has yet to be constructed measuring characteristics that can contribute to increased grit in adults. Existing scales that measure underlying qualities could be used; however, some aspects may not be relevant in the context of grit. That is, scales may measure attributes that are not directly applicable to improving one's level of grit. Furthermore, administering numerous questionnaires can overburden people, negatively impacting response rates and accuracy of answers (Olson, 2014). Without a single scale to measure behaviors thought to improve grit, it may be challenging to develop targeted programs and evaluate pre‐ and post‐training data. Thus, this study aimed to develop a scale to measure the psychological resources of grit. Another study is underway to evaluate the resulting scale's psychometric properties, and these results will be reported in due course.

## 3 METHOD

### 3.1 Study design

The Delphi technique was used to gain consensus on items to be included in the Grit Psychological Resources Scale (GPRS) from a panel of experts through three rounds of online surveys (Keeney et al., 2011). Following each round, responses were analyzed and used to construct subsequent surveys. Delphi studies have been used to gain consensus among experts on a wide range of matters such as defining concepts (e.g., Raine, 2006), developing guidelines (e.g., Cox et al., 2016), and scale development (e.g., Bauer et al., 2019). The technique has been used in various fields and disciplines, including nursing and health sciences (Sanna et al., 2022; Shimazaki et al., 2021). This study was approved by the University of Tasmania Social Sciences Human Research Ethics Committee, reference number S0020355 (H‐70103). The three‐round modified e‐Delphi was undertaken from July to October 2020.

### 3.2 Participants

The inclusion criteria for recruitment included being English‐speaking researchers, leaders and professionals over 18 years of age who are experts in grit and related constructs. A participant was considered an “expert” if they met at least one of the following criteria: (1) has published or presented on grit/related constructs; (2) has recent experience in a leadership role that involves building capabilities to improve performance; and (3) regularly uses grit/related constructs in a professional setting to help others set and achieve long‐term goals. Purposeful and snowball sampling was used for recruitment. Panel members were identified from reference lists in papers relating to grit and contacts in the first author's professional network on LinkedIn.

This study aimed to recruit 30 participants and have at least 23 complete all three rounds, which is considered adequate to produce reliable results (Akins et al., 2005). Recruitment was conducted during the COVID‐19 pandemic, and as such, people were experiencing changes to working conditions and employment. Accordingly, a large number of invitations (*n* = 201) were sent out to offset an anticipated low response rate. Potential participants were sent a personal email inviting them to participate and were encouraged to suggest other people from their networks. Invitations included an Internet link that directed participants to the first online survey in REDCap, a secure web‐based application that enables data collection for research studies. All participants self‐rated themselves against the inclusion criteria and had an opportunity to discuss any questions with the researchers before providing informed consent to participate.

### 3.3 Survey development

The construction of survey items involved an extensive literature review and input from colleagues with expertise in constructs of interest and scale development. First, using the model for the psychological resources of grit (Schimschal et al., 2022), definitions were developed to define how each psychological resource likely enabled individuals to attain their goals (see Table 1 for resources). Namely, interest was defined as a psychological resource for grit that enables an individual to explore and deepen their interests via the attributes of curiosity, self‐awareness, courage, and patience. Likewise, purpose enables an individual to make their interests more meaningful via the attributes of self‐determination, self‐concordance, self‐compassion, and emotional intelligence. Together, interest and purpose are resources that likely help people remain consistent with their focus and direction (i.e., demonstrate passion for long‐term goals). Practice was defined as a resource that enables an individual to persist with skill development via the attributes of hardiness, resilience, self‐efficacy, and flow. Similarly, hope enables an individual to develop and stick with goals via the attributes of goal orientation, growth mindset, optimism, and self‐regulation. Collectively, practice and hope are resources that may aid with overcoming setbacks and challenges (i.e., demonstrate perseverance for long‐term goals).

Second, definitions were developed for each attribute associated with the psychological resources of grit (see Table 1 for attributes). These definitions then informed the identification of behaviors that would likely be associated with scoring a low and a high score for each attribute. For example, an individual with low self‐regulation may struggle to change behavior, be distracted easily, and find it hard to adjust their pace. Conversely, an individual high in self‐regulation can change behavior, usually avoid distractions, and adjust their pace to be effective. Third, the behavioral descriptions were used to develop positively and negatively worded items. Care was taken to ensure the items were focused on behavior and were worded in an unbiased way using plain language. Existing scales were also reviewed to elicit insights on item wording, instructions, and response format. Initially, 200 positively and negatively worded items were developed, 50 for each psychological resource of grit. This number was then reduced to 100 positively worded items, 25 for each resource, following peer review and feedback.

### 3.4 Data collection and analysis

Surveys were set up in REDCap to ensure participants would be required to answer each question, thereby reducing the risk of missing data. Participants were asked to rate items according to the importance of inclusion in the scale using a 5‐point Likert scale (not important, slightly important, moderately important, important, very important). After each round, the number of items was reduced according to consensus and a predetermined level of reduction. This approach was chosen to create a short form survey with a low response burden that could be readily applied in a practice setting (Beatty et al., 2020). Consensus was based on the rank ordering of items according to mean rating scores. The number of items retained after each round was predetermined: 60 after Round 1, 40 after Round 2, and 20 after Round 3. A cut‐off score for retention was not used. Accordingly, after Round 1, items were rank ordered, and 60 items with the highest scores were selected (ranging from 3.7 to 4.7 for retained items). Following the same process, 40 items were retained after Round 2 (ranging from 3.5 to 4.5), and 20 after Round 3 (ranging from 3.9 to 4.5). When two items had the same mean rating score, the item with the smaller standard deviation was retained.

In Round 1, participants were asked to answer demographic questions for group analysis and rate 100 items. Responses were imported into Microsoft Excel for Mac (version 16.46), and descriptive statistics (mean and standard deviation, median and interquartile range, and frequencies) were generated to determine panel characteristics and group responses to items. Using the approach described above, 60 items, including at least two for each attribute, were retained for the second survey. In Round 2, participants were sent unique links to access the second survey and asked to re‐rate the items retained. Responses were analyzed and 40 items, including at least two for each attribute, were retained for the final survey. In Round 3, participants were asked to re‐rate the remaining items one final time. Following analysis, 20 items, including at least one for each attribute, were retained for scale construction. After each round, sensitivity tests were conducted to assess the potential for selection bias due to item reduction (Raine, 2006). Retaining a minimum number of items for each attribute did not appear to bias the results.

## 4 RESULTS

Of the 201 experts invited to participate, 30 completed the Round 1 survey (15% response rate), 24 completed Round 2 (80%), and 27 completed Round 3 (90%). In Delphi studies, participants are normally required to respond to all rounds. However, given the design of this study (reduction of items), participants were not required to complete every round, hence the higher participation rate for Round 3. Table 2 shows the panel characteristics in each round. In Round 1, the panel was multidisciplinary and comprised of both national and international experts working in academia and professional practice. The participants’ mean age was 49 years, and most had completed higher education, with 20% (*n* = 6) holding a Bachelor's degree, 30% (*n* = 9) a Master's degree, and 33.3% (*n* = 10) a Doctoral degree. Participants worked in a variety of industry sectors, including 43.3% (*n* = 13) from education and training and 30% (*n* = 9) from manufacturing. In subsequent rounds, the panel characteristics did not change markedly.

**TABLE 2:** Panel demographics

Characteristic	Round 1 (*N* = 30)	Round 2 (*N* = 24)	Round 3 (*N* = 27)
	*n* (%)	*n* (%)	*n* (%)
Gender			
Male	10 (33.3)	6 (25.0)	7 (25.9)
Female	20 (66.7)	18 (75.0)	20 (74.1)
Mean age in years (SD)	48.5 (11.8)	48.3 (12.6)	46.6 (10.1)
Highest qualification completed			
Certificate or diploma	2 (6.7)	2 (8.3)	2 (7.4)
Bachelor's degree	6 (20.0)	5 (20.8)	6 (22.2)
Graduate diploma or certificate	1 (3.3)	0 (0)	1 (3.7)
Bachelor honors degree	1 (3.3)	0 (0)	1 (3.7)
Master's degree	9 (30.0)	8 (33.3)	8 (29.6)
Doctoral degree	10 (33.3)	8 (33.3)	8 (29.6)
Other	1 (3.3)	1 (4.2)	1 (3.7)
Industry			
Education and training	13 (43.3)	9 (37.5)	11 (40.7)
Electricity, gas, water and waste services	1 (3.3)	1 (4.2)	1 (3.7)
Health care and social assistance	1 (3.3)	1 (4.2)	1 (3.7)
Manufacturing	9 (30.0)	7 (29.2)	9 (33.3)
Professional, scientific, and technical services	1 (3.3)	1 (4.2)	1 (3.7)
Retail trade	1 (3.3)	1 (4.2)	1 (3.7)
Other	4 (13.3)	4 (16.7)	3 (11.1)

*Note*: Percentages may not total 100 due to rounding.

Table 3 presents mean rating scores for each item across all rounds. After each round, the number of items was progressively reduced from 100 to 20 according to group responses. After Round 1, 60 items were retained for Round 2, 15 for each resource of grit. Mean rating scores for retained items ranged from 3.7 to 4.7 out of 5, with 41 (68%) scoring ≥4. After Round 2, 40 items were retained for Round 3, 10 for each resource of grit. Mean rating scores for retained items ranged from 3.5 to 4.5, with 22 (55%) scoring ≥4. After Round 3, 20 items were retained to construct the final 20‐item scale with mean rating scores ranging from 3.9 to 4.5, with 17 (85%) scoring ≥4. The scale has five items linked to each resource, with at least one item for each attribute (16 in total) thought to strengthen grit. A sensitivity analysis conducted after each round demonstrated that retaining a minimum number of items for each attribute (two after Round 1 and Round 2, and one after Round 3) would not have resulted in selection bias. Median and interquartile ranges are also reported, although these were not used to remove items after each round. Median scores in Round 1 ranged from 3.0 to 5.0, in Round 2 they ranged from 4.0 to 5.0, and in the final scale median scores ranged from 4.0 to 5.0.

**TABLE 3:** Mean and median rating scores for items

Item	Attribute	Round 1		Round 2		Round 3	
		M (SD)	Mdn (IQR)	M (SD)	Mdn (IQR)	M (SD)	Mdn (IQR)
Interest							
I can usually find ways to remain committed^a^	—	4.4 (0.7)	5.0 (1.0)	3.8 (0.8)	4.0 (1.0)	RR2	RR2
I often look for new experiences^b^	Curiosity	3.4 (1.3)	4.0 (1.8)	RR1	RR1	RR1	RR1
I enjoy having new experiences	Curiosity	3.4 (1.4)	4.0 (3.0)	RR1	RR1	RR1	RR1
I seek out activities that are demanding or uncertain	Curiosity	3.4 (1.0)	3.5 (1.0)	RR1	RR1	RR1	RR1
I am slow to lose interest in things	Curiosity	3.5 (1.2)	3.5 (1.8)	RR1	RR1	RR1	RR1
I usually stay engaged with activities	Curiosity	3.9 (1.0)	4.0 (2.0)	3.8 (0.9)	4.0 (1.0)	3.7 (1.0)	4.0 (0.5)
**I am always looking to learn new things**	**Curiosity**	**4.2 (1.1)**	**4.5 (1.0)**	**4.1 (1.0)**	**4.0 (1.0)**	**4.2 (0.8)**	**4.0 (1.0)**
I often use reflection to inform my decisions^b^	Self‐awareness	4.0 (1.3)	4.0 (1.0)	3.8 (1.2)	4.0 (2.0)	RR2	RR2
I often reflect on my experiences	Self‐awareness	3.8 (1.2)	4.0 (2.0)	3.9 (1.1)	4.0 (2.0)	3.9 (1.2)	4.0 (2.0)
I think about the reasons for my choices	Self‐awareness	3.9 (1.2)	4.0 (1.8)	3.6 (1.0)	4.0 (1.0)	RR2	RR2
I use insights to learn from my experiences	Self‐awareness	3.9 (1.2)	4.0 (2.0)	3.8 (0.9)	4.0 (1.0)	RR2	RR2
I use insights to adapt my behavior.	Self‐awareness	4.2 (1.1)	4.5 (1.0)	4.0 (0.9)	4.0 (0.3)	3.7 (1.0)	4.0 (1.0)
**I am usually aware of my behavior**	**Self‐awareness**	**3.8 (1.2)**	**4.0 (2.0)**	**4.0 (1.0)**	**4.0 (1.3)**	**3.9 (1.0)**	**4.0 (2.0)**
I am willing to do things that involve change^b^	Courage	4.0 (1.2)	4.0 (1.0)	4.0 (1.0)	4.0 (1.0)	4.1 (1.2)	4.0 (1.5)
I am comfortable taking risks	Courage	3.5 (1.1)	4.0 (1.0)	RR1	RR1	RR1	RR1
I like to try new things	Courage	3.5 (1.2)	4.0 (1.0)	RR1	RR1	RR1	RR1
**I can work outside my comfort zone**	**Courage**	**4.1 (1.0)**	**4.0 (1.0)**	**4.1 (0.9)**	**4.0 (1.0)**	**4.1 (0.8)**	**4.0 (1.0)**
The possibility of failure does not scare me	Courage	3.5 (0.9)	3.5 (1.0)	RR1	RR1	RR2	RR2
I can be brave when required	Courage	3.7 (1.1)	4.0 (1.0)	3.9 (1.2)	4.0 (1.3)	3.8 (1.1)	4.0 (2.0)
**I am able to tolerate uncertainty with the way forward** ^**b**^	**Patience**	**4.2 (1.1)**	**5.0 (1.0)**	**4.0 (1.0)**	**4.0 (1.0)**	**4.1 (0.8)**	**4.0 (1.0)**
I am patient when exploring new ideas	Patience	3.4 (1.1)	3.0 (1.0)	RR1	RR1	RR1	RR1
I am patient when learning new skills	Patience	3.7 (0.9)	4.0 (1.0)	RR1	RR1	RR1	RR1
I remain calm when learning is hard	Patience	3.6 (1.1)	4.0 (1.0)	RR1	RR1	RR1	RR1
I am able to spend time on complex problems	Patience	3.8 (0.9)	4.0 (0.8)	3.8 (1.0)	4.0 (1.0)	RR2	RR2
**I usually persist with learning**	**Patience**	**4.1 (0.9)**	**4.0 (1.0)**	**4.3 (0.8)**	**4.0 (1.0)**	**4.3 (0.8)**	**4.0 (1.0)**
Purpose							
**I am committed to my goals** ^**a**^	—	**4.7 (0.6)**	**5.0 (0.8)**	**4.5 (0.6)**	**5.0 (1.0)**	**4.3 (1.0)**	**4.0 (1.0)**
I have control over my direction in life^b^	Self‐determination	4.2 (1.2)	5.0 (1.0)	3.9 (1.0)	4.0 (0.3)	3.8 (1.1)	4.0 (2.0)
I often select my own activities	Self‐determination	3.7 (1.1)	4.0 (1.0)	RR1	RR1	RR1	RR1
I work hard on my goals	Self‐determination	4.4 (0.8)	4.5 (1.0)	3.9 (0.9)	4.0 (2.0)	RR2	RR2
I have goals to achieve something important	Self‐determination	4.4 (1.0)	5.0 (1.0)	3.9 (1.1)	4.0 (1.3)	4.2 (0.9)	4.0 (1.0)
I have goals that will make a difference	Self‐determination	4.1 (1.1)	4.5 (1.0)	3.8 (1.0)	4.0 (1.0)	RR2	RR2
**My goals provide purposeful direction**	**Self‐determination**	**4.4 (1.0)**	**5.0 (1.0)**	**4.1 (0.9)**	**4.0 (1.0)**	**4.5 (0.7)**	**5.0 (1.0)**
**I pursue activities that are of value to me** ^**b**^	**Self‐concordance**	**4.3 (0.9)**	**5.0 (1.0)**	**4.1 (0.9)**	**4.0 (1.0)**	**4.2 (0.7)**	**4.0 (1.0)**
I work hard on things because I value them	Self‐concordance	4.5 (0.7)	5.0 (1.0)	4.2 (0.6)	4.0 (1.0)	4.2 (1.0)	4.0 (1.0)
I pursue activities because I choose to	Self‐concordance	3.8 (1.0)	4.0 (0.8)	3.8 (1.0)	4.0 (2.0)	RR2	RR2
I stick with activities to gain experience	Self‐concordance	3.4 (1.0)	3.5 (1.0)	RR1	RR1	RR1	RR1
I persist with goals to stay motivated	Self‐concordance	3.4 (1.1)	3.0 (1.0)	RR1	RR1	RR1	RR1
I strive toward goals to improve	Self‐concordance	4.0 (0.8)	4.0 (0.8)	3.5 (1.1)	4.0 (1.0)	RR2	RR2
I am kind to myself when things go wrong^b^	Self‐compassion	3.7 (1.1)	4.0 (1.8)	3.7 (1.1)	4.0 (2.0)	3.8 (1.0)	4.0 (0.5)
**I try to learn from bad experiences**	**Self‐compassion**	**3.9 (1.1)**	**4.0 (1.8)**	**3.5 (1.1)**	**4.0 (1.0)**	**4.0 (0.9)**	**4.0 (0.5)**
I am accepting of choices that do not work out	Self‐compassion	3.5 (1.1)	4.0 (1.0)	RR1	RR1	RR1	RR1
My direction and goals are evolving	Self‐compassion	3.5 (1.3)	4.0 (3.0)	RR1	RR1	RR1	RR1
I go easy on myself with setbacks	Self‐compassion	3.2 (1.1)	3.0 (1.8)	RR1	RR1	RR1	RR1
I am gentle with myself when I fail at something	Self‐compassion	3.4 (1.2)	4.0 (1.0)	RR1	RR1	RR1	RR1
I manage my emotions to maintain direction^b^	Emotional Intelligence	4.0 (0.8)	4.0 (1.5)	3.9 (1.0)	4.0 (2.0)	3.9 (0.9)	4.0 (1.0)
I usually notice when my emotions are distracting me	Emotional Intelligence	3.7 (1.1)	4.0 (1.0)	RR1	RR1	RR1	RR1
I am aware of how I feel about different activities	Emotional Intelligence	3.5 (1.2)	4.0 (1.8)	RR1	RR1	RR1	RR1
**I can overcome negative emotions when things are tough**	**Emotional Intelligence**	**4.1 (0.8)**	**4.0 (1.0)**	**3.9 (1.0)**	**4.0 (1.3)**	**4.0 (1.0)**	**4.0 (1.5)**
I do not let my feelings stop me from working	Emotional Intelligence	3.8 (1.1)	4.0 (2.0)	3.8 (0.9)	4.0 (0.3)	RR2	RR2
My emotions provide insights that help me to stay on track	Emotional Intelligence	3.7 (1.0)	4.0 (1.0)	RR1	RR1	RR1	RR1
Practice							
I usually practice to achieve a specific goal^a^	—	3.8 (1.0)	4.0 (2.0)	3.6 (1.0)	4.0 (1.0)	RR2	RR2
I work hard to develop skills^b^	Hardiness	4.2 (0.7)	4.0 (1.0)	4.0 (1.0)	4.0 (1.3)	4.0 (0.7)	4.0 (1.0)
In general I stay composed when pressured to learn	Hardiness	3.7 (1.2)	4.0 (0.8)	3.6 (0.9)	4.0 (1.0)	RR2	RR2
I generally have control over developing expertise	Hardiness	3.6 (0.9)	4.0 (1.0)	RR1	RR1	RR1	RR1
Extra practice helps me to improve	Hardiness	4.0 (1.0)	4.0 (1.0)	3.9 (1.1)	4.0 (1.3)	RR2	RR2
**As a rule I keep trying until I figure things out**	**Hardiness**	**4.3 (0.8)**	**4.5 (1.0)**	**4.2 (0.9)**	**4.0 (1.0)**	**4.1 (0.9)**	**4.0 (1.0)**
I usually remain positive when experiencing difficulties	Hardiness	3.9 (0.6)	4.0 (0.0)	3.8 (1.1)	4.0 (0.3)	RR2	RR2
**I usually rebound after difficulties** ^**b**^	**Resilience**	**4.1 (0.8)**	**4.0 (1.0)**	**4.2 (0.6)**	**4.0 (1.0)**	**4.2 (0.7)**	**4.0 (1.0)**
**I am good at dealing with challenges**	**Resilience**	**4.3 (0.7)**	**4.0 (1.0)**	**4.2 (0.9)**	**4.0 (1.0)**	**4.2 (0.8)**	**4.0 (1.0)**
Somehow or other I overcome setbacks	Resilience	4.0 (1.0)	4.0 (1.0)	4.1 (0.9)	4.0 (1.0)	RR2	RR2
I am quick to recover from failure	Resilience	4.0 (1.0)	4.0 (1.8)	4.1 (0.7)	4.0 (1.0)	3.9 (0.7)	4.0 (1.0)
Past adversity has made me stronger	Resilience	4.1 (1.1)	4.0 (1.0)	4.2 (0.9)	4.0 (1.0)	4.0 (0.7)	4.0 (0.5)
I use stress to my advantage	Resilience	3.3 (1.1)	3.0 (1.0)	RR1	RR1	RR1	RR1
**I have the capabilities to be successful** ^**b**^	**Self‐efficacy**	**3.9 (1.0)**	**4.0 (2.0)**	**3.7 (0.9)**	**4.0 (1.0)**	**3.9 (1.1)**	**4.0 (1.5)**
My confidence supports me to achieve my goals	Self‐efficacy	3.6 (1.2)	4.0 (1.0)	RR1	RR1	RR1	RR1
My confidence supports me to solve problems	Self‐efficacy	3.6 (1.1)	4.0 (1.0)	RR1	RR1	RR1	RR1
I like to set challenging goals	Self‐efficacy	3.9 (0.8)	4.0 (1.0)	3.6 (1.0)	4.0 (1.0)	3.6 (1.1)	4.0 (1.0)
If I fail it is rarely due to my abilities	Self‐efficacy	2.7 (1.0)	3.0 (1.0)	RR1	RR1	RR1	RR1
Extra effort usually gets me out of trouble	Self‐efficacy	3.4 (1.0)	3.5 (1.0)	RR1	RR1	RR1	RR1
I am able to work effectively with ease^b^	Flow	3.3 (1.1)	3.0 (1.8)	RR1	RR1	RR1	RR1
I have goals that stretch me	Flow	4.1 (0.8)	4.0 (1.0)	3.6 (0.8)	4.0 (1.0)	3.9 (0.9)	4.0 (1.5)
**What I want to achieve is clear to me**	**Flow**	**4.2 (0.8)**	**4.0 (1.0)**	**3.7 (1.0)**	**4.0 (1.3)**	**3.9 (1.1)**	**4.0 (2.0)**
I have the skills to perform seamlessly	Flow	3.1 (1.0)	3.0 (2.0)	RR1	RR1	RR1	RR1
I usually perform without worrying about failure	Flow	3.4 (1.1)	4.0 (1.0)	RR1	RR1	RR1	RR1
I get regular feedback on my performance	Flow	3.6 (1.1)	4.0 (1.8)	RR1	RR1	RR1	RR1
Hope							
I actively use various tactics to persist with my goals^a^	—	4.1 (1.0)	4.0 (1.0)	3.9 (0.9)	4.0 (1.3)	RR2	RR2
I mostly pursue goals to improve my capabilities^b^	Goal orientation	3.2 (1.2)	3.0 (1.0)	RR1	RR1	RR1	RR1
Setbacks motivate me to work harder	Goal orientation	3.8 (0.7)	4.0 (1.0)	3.7 (0.8)	4.0 (1.0)	RR2	RR2
I enjoy challenging goals because they help me learn	Goal orientation	4.2 (0.9)	4.0 (1.0)	3.9 (0.8)	4.0 (0.3)	3.8 (1.0)	4.0 (1.5)
I enjoy activities that stretch my capabilities	Goal orientation	4.2 (0.9)	4.0 (1.0)	4.0 (0.9)	4.0 (0.3)	3.9 (1.0)	4.0 (1.0)
**Continuous improvement is important to me**	**Goal orientation**	**4.3 (0.8)**	**5.0 (1.0)**	**4.0 (0.8)**	**4.0 (1.0)**	**4.0 (0.8)**	**4.0 (0.5)**
**Feedback helps me to improve**	**Goal orientation**	**4.0 (1.1)**	**4.0 (1.8)**	**4.0 (1.0)**	**4.0 (1.0)**	**4.1 (1.0)**	**4.0 (1.5)**
Most of my success is a result of hard work^b^	Growth mindset	3.9 (1.0)	4.0 (2.0)	3.8 (1.0)	4.0 (2.0)	RR2	RR2
I can change my characteristics if I want to	Growth mindset	3.3 (1.1)	3.0 (1.0)	RR1	RR1	RR1	RR1
**I can improve my capabilities with effort**	**Growth mindset**	**4.2 (0.7)**	**4.0 (1.0)**	**4.2 (0.8)**	**4.0 (1.0)**	**4.1 (0.9)**	**4.0 (1.5)**
I have the potential to achieve great things	Growth mindset	4.4 (0.9)	5.0 (1.0)	3.9 (1.0)	4.0 (1.0)	4.0 (0.7)	4.0 (0.5)
I use failure to keep getting better	Growth mindset	3.7 (0.8)	4.0 (1.0)	RR1	RR1	RR1	RR1
I am open about my shortcomings	Growth mindset	3.8 (1.2)	4.0 (2.0)	3.6 (1.1)	4.0 (1.3)	RR2	RR2
I usually resolve problems that slow me down^b^	Optimism	3.7 (1.0)	4.0 (1.0)	RR1	RR1	RR1	RR1
I seldom take setbacks personally	Optimism	3.2 (1.1)	3.0 (2.0)	RR1	RR1	RR1	RR1
I can usually get my goals back on track	Optimism	4.1 (0.6)	4.0 (0.0)	3.9 (0.9)	4.0 (1.3)	3.9 (0.9)	4.0 (1.5)
**I find most problems can be solved**	**Optimism**	**4.0 (0.9)**	**4.0 (1.0)**	**3.7 (1.2)**	**4.0 (2.0)**	**4.0 (0.9)**	**4.0 (0.5)**
I usually take risks into consideration	Optimism	3.4 (1.2)	3.0 (1.0)	RR1	RR1	RR1	RR1
I use prior learning to make future plans	Optimism	3.7 (1.0)	4.0 (1.8)	RR1	RR1	RR1	RR1
I am able to control my behavior for goal pursuit^b^	Self‐regulation	4.0 (1.1)	4.0 (1.8)	3.8 (1.1)	4.0 (0.3)	RR2	RR2
When required, I can change my behavior	Self‐regulation	3.8 (1.1)	4.0 (1.8)	RR1	RR1	RR1	RR1
I usually avoid distractions	Self‐regulation	3.0 (1.0)	3.0 (2.0)	RR1	RR1	RR1	RR1
**I can stay focused on the big‐picture**	**Self‐regulation**	**4.3 (0.8)**	**4.0 (1.0)**	**4.2 (1.0)**	**4.0 (1.0)**	**4.2 (0.9)**	**4.0 (1.0)**
I frequently monitor my progress	Self‐regulation	4.2 (0.9)	4.0 (1.0)	3.9 (1.0)	4.0 (2.0)	4.0 (0.9)	4.0 (1.0)
I can alter my pace to be more effective	Self‐regulation	3.8 (0.9)	4.0 (0.0)	RR1	RR1	RR1	RR1

*Note*: Bolded items retained for the final scale. Expert ratings reflect the importance of inclusion in the GPRS (1 = not important; 2 = slightly important; 3 = moderately important; 4 = important; 5 = very important). IQR, interquartile range; Mdn, median; RR1, item removed after Round 1; RR2, item removed after Round 2.

^a^ Overarching items for the resource.

^b^ Overarching items for the attribute.

Table 4 presents the number of items and mean rating scores grouped by resource and attribute. In Round 1, there were 25 items for each resource of grit (interest, purpose, practice, and hope). Of the 25 items, there was one overarching item for the resource and six items for each linked attribute (e.g., curiosity, self‐awareness, courage, and patience for the resource of interest). The number of items for each resource was reduced to 15 items in Round 2, 10 in Round 3, and five in the final scale. After each round, the number of items retained for each attribute was reduced according to expert ratings. In Round 2, there was a higher proportion of items linked to self‐awareness, self‐determination, hardiness, resilience, and goal orientation, which continued in Round 3, with the exception of hardiness.

**TABLE 4:** Number of items and mean rating scores grouped by resource and attribute

Resource/attribute	Number of items linked to resource/attribute				Mean rating scores			
	Round 1	Round 2	Round 3	Final scale	Round 1	Round 2	Round 3	Final scale
	*n*	*n*	*n*	*n*	M (SD)	M (SD)	M (SD)	M (SD)
Interest	25	15	10	5	3.8 (1.1)	3.9 (1.0)	4.0 (1.0)	4.1 (0.8)
Overarching (Interest)	1	1	0	0	4.4 (0.7)	3.8 (0.8)	NA	NA
Curiosity	6	2	2	1	3.6 (1.2)	3.9 (0.9)	4.0 (0.9)	4.2 (0.8)
Self‐awareness	6	6	3	1	3.9 (1.2)	3.8 (1.0)	3.8 (1.1)	3.9 (1.0)
Courage	6	3	3	1	3.7 (1.1)	4.0 (1.0)	4.0 (1.0)	4.1 (0.8)
Patience	6	3	2	2	3.8 (1.0)	4.0 (0.9)	4.2 (0.8)	4.2 (0.8)
Purpose	25	15	10	5	3.9 (1.0)	3.9 (1.0)	4.1 (0.9)	4.2 (0.9)
Overarching (Purpose)	1	1	1	1	4.7 (0.6)	4.5 (0.6)	4.3 (1.0)	4.3 (1.0)
Self‐concordance	6	4	2	1	3.9 (0.9)	3.9 (0.9)	4.2 (0.9)	4.2 (0.7)
Self‐determination	6	5	3	1	4.2 (1.0)	3.9 (1.0)	4.2 (0.9)	4.5 (0.7)
Self‐compassion	6	2	2	1	3.5 (1.2)	3.6 (1.1)	3.9 (0.9)	4.0 (0.9)
Emotional Intelligence	6	3	2	1	3.8 (1.0)	3.8 (1.0)	4.0 (0.9)	4.0 (1.0)
Practice	25	15	10	5	3.8 (1.0)	3.9 (0.9)	4.0 (0.8)	4.1 (0.9)
Overarching (Practice)	1	1	0	0	3.8 (1.0)	3.6 (1.0)	NA	NA
Hardiness	6	5	2	1	4.0 (0.9)	3.9 (1.0)	4.0 (0.8)	4.1 (0.9)
Resilience	6	5	4	2	4.0 (0.9)	4.2 (0.8)	4.1 (0.7)	4.2 (0.8)
Self‐efficacy	6	2	2	1	3.5 (1.0)	3.6 (0.9)	3.7 (1.1)	3.9 (1.1)
Flow	6	2	2	1	3.6 (1.0)	3.6 (0.9)	3.9 (1.0)	3.9 (1.1)
Hope	25	15	10	5	3.9 (1.0)	3.9 (1.0)	4.0 (0.9)	4.1 (0.9)
Overarching (Hope)	1	1	0	0	4.1 (1.0)	3.9 (0.9)	NA	NA
Goal orientation	6	5	4	2	3.9 (0.9)	3.9 (0.9)	4.0 (0.9)	4.1 (0.9)
Growth mindset	6	4	2	1	3.9 (1.0)	3.9 (1.0)	4.1 (0.8)	4.1 (0.9)
Optimism	6	2	2	1	3.7 (1.0)	3.8 (1.0)	3.9 (0.9)	4.0 (0.9)
Self‐regulation	6	3	2	1	3.8 (1.0)	4.0 (1.0)	4.1 (0.9)	4.2 (0.9)

Mean rating scores for resources across rounds ranged from 3.8 to 4.1, with an average of 4.1 in the final scale, indicating a strong agreement. Purpose was the only resource for which an overarching item was retained after Round 2. The mean level of agreement for attributes ranged from 3.9 to 4.5 in the final scale, with self‐awareness, self‐efficacy, and flow receiving slightly lower ratings. Items linked to overarching purpose and self‐determination received the highest ratings of 4.3 and 4.5, respectively. Overall, the mean rating scores for resources and attributes increased with item reduction after each round.

Table 5 presents the frequency of rating responses in Round 3 for items included in the final scale. Agreement was reached for all items (at least 51% of participants rating as 4 or 5). Items linked to patience and overarching purpose received the highest level of agreement, with 24 out of 27 (88.9%) participants rating as 4 or 5. A high level of agreement was also reached with items for courage, self‐determination, goal orientation, and self‐regulation: 23 (85.2%) participants rating as 4 or 5. Items linked to self‐awareness and flow had the lowest level of agreement, with 18 (66.7%) and 17 (63.0%) participants rating as 4 or 5, respectively. The proportion of participants who rated items as not important (rating 1) or slightly important (rating 2) was very low.

**TABLE 5:** Frequency of rating responses in Round 3 for items included in the final scale

Item	Attribute	Frequency of rating responses			
		Rating 1	Rating 2	Rating 3	Rating ≥4
		*n* (%)	*n* (%)	*n* (%)	*n* (%)
Interest					
I am always looking to learn new things	Curiosity	0 (0)	1 (3.7)	4 (14.8)	22 (81.5)
I am usually aware of my behavior	Self‐awareness	1 (3.7)	1 (3.7)	7 (25.9)	18 (66.7)
I can work outside my comfort zone	Courage	0 (0)	1 (3.7)	3 (11.1)	23 (85.2)
I usually persist with learning	Patience	0 (0)	1 (3.7)	2 (7.4)	24 (88.9)
I am able to tolerate uncertainty with the way forward	Patience	0 (0)	1 (3.7)	4 (14.8)	22 (81.5)
Purpose					
I am committed to my goals	—	1 (3.7)	1 (3.7)	1 (3.7)	24 (88.9)
I pursue activities that are of value to me	Self‐concordance	0 (0)	0 (0)	5 (18.5)	22 (81.5)
My goals provide purposeful direction	Self‐determination	0 (0)	0 (0)	4 (14.8)	23 (85.2)
I try to learn from bad experience	Self‐compassion	1 (3.7)	0 (0)	5 (18.5)	21 (77.8)
I can overcome negative emotions when things are tough	Emotional Intelligence	0 (0)	3 (11.1)	4 (14.8)	20 (74.1)
Practice					
As a rule I keep trying until I figure things out	Hardiness	0 (0)	2 (7.4)	4 (14.8)	21 (77.8)
I am good at dealing with challenges	Resilience	0 (0)	0 (0)	6 (22.2)	21 (77.8)
I usually rebound after difficulties	Resilience	0 (0)	0 (0)	5 (18.5)	22 (81.5)
I have the capabilities to be successful	Self‐efficacy	2 (7.4)	1 (3.7)	4 (14.8)	20 (74.1)
What I want to achieve is clear to me	Flow	1 (3.7)	2 (7.4)	7 (25.9)	17 (63.0)
Hope					
Continuous improvement is important to me	Goal orientation	1 (3.7)	0 (0)	3 (11.1)	23 (85.2)
Feedback helps me to improve	Goal orientation	1 (3.7)	0 (0)	6 (22.2)	20 (74.1)
I can improve my capabilities with effort	Growth mindset	0 (0)	1 (3.7)	6 (22.2)	20 (74.1)
I find most problems can be solved	Optimism	1 (3.7)	1 (3.7)	3 (11.1)	22 (81.5)
I can stay focused on the big‐picture	Self‐regulation	1 (3.7)	0 (0)	3 (11.1)	23 (85.2)

*Note*: Expert ratings of items retained for the final scale (1 = not important; 2 = slightly important; 3 = moderately important; 4 = important; 5 = very important).

## 5 DISCUSSION

This Delphi study used ratings from a panel of 24 experts to identify items for inclusion in the GPRS, a scale to measure the psychological resources of grit. After three rounds, strong agreement was achieved for 20 of the 100 items proposed for the scale. The mean rating scores for psychological resources of interest, purpose, practice, and hope were similar across all rounds, indicating participants viewed these assets as equally important for grit (Duckworth, 2016). Furthermore, participants rated items related to each psychological resource as important for inclusion in the final scale. Only the overarching item for purpose (“I am committed to my goals”) was retained. Overarching items for the psychological resources of interest (“I can usually find ways to remain committed”), practice (“I usually practice to achieve a specific goal”), and hope (“I actively use various tactics to persist with my goals”) had good agreement in Round 1 but were dropped in Round 2. The overarching item for interest and items linked to curiosity saw the largest change with a 0.6‐point difference. This suggests participants may have had some difficulty rating one item higher than another. Additionally, participants may not have been able to differentiate between overarching items related to a psychological resource and those relating to associated attributes.

After the first round, a preference emerged for one to two attributes linked to each resource. These results largely persisted in the final round. Specifically, no items for self‐awareness were removed after the first round. Similar results were seen for four other attributes. Only one item each was removed after Round 1 for self‐determination, hardiness, resilience, and goal orientation. The preference for items linked to resilience and goal orientation persisted into the third round, suggesting participants viewed these attributes as more important for grit. Alternatively, the items may have resonated more strongly with participants, especially since the survey was conducted during the COVID‐19 pandemic. That is, job losses and changes to working conditions required many people to retrain in new industries and work flexibly, which in turn likely required increased resilience and goal orientation (ILO and OECD, 2020).

Items for some attributes were viewed less positively after the first round. For example, four items each for curiosity, self‐compassion, self‐efficacy, flow, and optimism were removed after the first round. As with those viewed more positively, some items linked to these five attributes may have resonated less with participants. Alternatively, participants had less understanding of or experience working with these attributes. Nevertheless, items retained after the final round had median scores of ≥4.0, indicating that there was a good level of agreement for items at both the resource and attribute level, which lends support for the structure of the final scale.

### 5.1 Strengths and limitations

A panel of experts reached consensus on important items for inclusion in the GPRS. The inclusion criteria for participation in this study enabled the research team to gather expert feedback from an academic and practitioner standpoint. Of the 30 participants who began the study, only six did not complete all rounds, enabling reliable results (Akins et al., 2005). The participation and response rates were positive, given that this study was conducted during a global pandemic, which may have limited communication due to forced leave and severances (Hales, 2021). Conversely, working conditions may have provided people with more time and flexibility to participate in the study (de Lucas Ancillo et al., 2021). One limitation of the study is the similar mean rating scores seen across items making it hard to select items for reduction. These results could suggest that all of the items being considered were thought to be important. Alternatively, participants may have all had a very positive disposition, making it more difficult to rate some items as more important than others. Nonetheless, the good level of agreement gave support to retaining a minimum number of items for each attribute through the process and, therefore, to the final structure of the GPRS.

### 5.2 Practical implications

The 20‐item GPRS resulting from this Delphi study has several practical implications. The scale could be used as a tool to identify opportunities to strengthen psychological resources that enable consistent interest in and perseverance with ideas, projects, and goals. As the context will likely influence the extent to which an individual accrues and applies the psychological resources of grit, several variations of scale instructions have been developed so users can get more accurate results. For example, an individual may be interested in their current psychological resources in general. Alternatively, information may be wanted in an employment or sports context when working toward specific individual or team projects and goals. Thus, the separate scores for interest, purpose, practice, and hope, as well as the total score, may provide insights into the reasons for satisfaction and engagement in different areas of life. Scores could also provide leaders and those responsible for developing individual and team capabilities with feedback on potential training needs. Depending on the nature of work and psychological resources of grit potentially contributing more or less to goal achievement, various interventions could be considered, such as self‐directed learning and professional coaching. Finally, the GPRS could be used together with other reliable existing scales, such as scales for grit (Duckworth et al., 2007), passion (Vallerand et al., 2003), and persistence (Howard & Crayne, 2019), to enhance needs analysis further.

## 6 CONCLUSION

Utilizing a national and international panel of experts in grit and related constructs, this study obtained a consensus on items to include in the GPRS. It is expected that this scale will provide a wide variety of professionals across various industries an effective tool to assess the psychological resources of grit at an individual or team level and in different contexts. Additionally, professionals could use the scale at different time points to identify the changing needs of individuals and businesses. Future research could test the reliability and validity of the scale.

## Data Availability

Data are available upon reasonable request.
